# CPLX1 is a novel prognostic biomarker in CRC correlating with immunotherapy resistance and ferroptosis

**DOI:** 10.3389/fimmu.2025.1589423

**Published:** 2025-07-09

**Authors:** Canyu Liu, Qiujun Liu, Yuanhao Lv, Tingmin Chang, Shiyi Song, Yuang Ding, Jiateng Zhong, Yanxuan Liu

**Affiliations:** ^1^ Department of Digestive Endoscopy Center, The First Affiliated Hospital of Xinxiang Medical University, Xinxiang, China; ^2^ Department of Endocrinology, The First Affiliated Hospital of Xinxiang Medical University, Xinxiang, China; ^3^ Department of Pathology, Xinxiang Medical University, Xinxiang, China; ^4^ Department of Pathology, The First Affiliated Hospital of Xinxiang Medical University, Xinxiang, China; ^5^ Xinxiang Key Laboratory of Precision Diagnosis and Treatment for Colorectal Cancer, Xinxiang First People’s Hospital, Xinxiang, China; ^6^ Xinxiang Engineering Technology Research Center of Digestive Tumor Molecular Diagnosis, Xinxiang Medical University, Xinxiang, China; ^7^ Henan Province Engineering Technology Research Center of Tumor Diagnostic Biomarkers and RNA Interference Drugs, The Third Affiliated Hospital of Xinxiang Medical University, Xinxiang, China; ^8^ Department of Medical Genetics, The First Affiliated Hospital of Xinxiang Medical University, Xinxiang, China

**Keywords:** CPLX1, biomarker, CRC, tumor-immune infiltration, ferroptosis

## Abstract

**Background:**

Colorectal cancer (CRC) remains a predominant contributor to cancer-related mortality globally, with its resistance to immunotherapeutic strategies presenting a formidable challenge in patient management. Recent investigations have illuminated the prospective involvement of ferroptosis, a regulated form of cell death, in both cancer progression and the development of resistance to therapeutic interventions.

**Objective:**

This study aims to elucidate the prognostic significance of CPLX1 in CRC, specifically its correlation with immunotherapy resistance and its association with ferroptosis, thereby contributing to a deeper understanding of tumor biology and therapeutic vulnerability.

**Methods:**

We conducted an integrative analysis of RNA-seq datasets from the TCGA-COAD and TCGA-READ projects, along with the GEO GSE156451 dataset, to discern differentially expressed genes. Expression levels of CPLX1 were evaluated utilizing the TIMER 2.0 database, and survival analyses were performed via Kaplan-Meier plots and Cox regression modeling to assess prognostic implications. Additionally, mutational analyses through cBioPortal and COSMIC datasets were employed to identify CPLX1 mutations in COAD. Co-expression and functional enrichment analyses, alongside Gene Set Enrichment Analysis (GSEA), were also conducted to delineate pathways impacted by CPLX1.

**Results:**

Our findings indicate that high expression levels of CPLX1 are significantly correlated with poor prognostic outcomes in CRC patients. Through immune infiltration analyses employing ssGSEA, we observed notable associations between CPLX1 expression and specific immune cell populations. Furthermore, the interaction between CPLX1 and ferroptosis-related genes suggests a potential mechanistic linkage that could underpin therapeutic resistance.

**Conclusion:**

CPLX1 is identified as a novel prognostic biomarker in CRC, exhibiting clear correlations with both immunotherapy resistance and ferroptosis. These findings indicate that targeting CPLX1 may provide novel therapeutic strategies to ameliorate treatment resistance in CRC.

## 1 Introduction

Colorectal cancer (CRC) represents a significant global health challenge, being one of the leading causes of cancer-related morbidity and mortality (1). Current treatment modalities for CRC, including surgical resection, chemotherapy, and targeted therapies, have shown varying degrees of efficacy; however, the emergence of resistance to immunotherapy and the complex interplay of tumor microenvironment factors, such as ferroptosis, remain critical hurdles in improving patient outcomes (2). Despite advancements in understanding the molecular underpinnings of CRC, there exists a notable gap in identifying reliable prognostic biomarkers that can predict treatment responses and guide therapeutic strategies.

CPLX1 (Complexin I) is a small synaptic protein that plays an important role mainly in the central nervous system and is involved in the release of neurotransmitters and the functional regulation of synapses. CPLX1 regulates the anchoring, preactivation, and fusion processes of synaptic vesicles through its interaction with the SNARE complex, thus playing a key role in neural signalin (3). In recent years, it has been found that CPLX1 exhibits aberrant expression in a variety of neurodegenerative diseases and psychiatric disorders, which has attracted extensive attention from the scientific community (4). In addition, mutations or deletions in CPLX1 have been associated with a variety of neurobehavioral deficits, demonstrating its importance in neurological health (3). In recent years, the relationship between CPLX1 and cancer has been gradually emphasized. Studies have shown that CPLX1 may influence the behavior of tumor cells in the cancer microenvironment and participate in tumorigenesis and progression. For example, in a cancer-induced bone pain model, changes in CPLX1 expression were closely associated with the occurrence of pain, suggesting that CPLX1 may play an important role in the mechanism of cancer-related pain (5). Tanaka et al. demonstrated that CPLX1 may be a biomarker and therapeutic target for gastric cancer recurrence by transcriptomics and ex vivo experiments on surgically resected specimens from 16 UICC stage III gastric cancer patients who underwent radical gastrectomy and adjuvant oral fluoropyrimidine monotherapy (6). In addition, CPLX1 may serve as a biomarker in certain types of cancer, providing new ideas for early diagnosis and prognostic assessment of cancer (7).

This study employs a comprehensive bioinformatics approach to investigate the role of CPLX1 as a novel prognostic biomarker in colorectal cancer (CRC), particularly in relation to immunotherapy resistance and ferroptosis. The methodology integrates RNA-seq data from the TCGA and GEO databases, allowing for a robust analysis of gene expression patterns across various cancer types. The advantages of this approach lie in its ability to leverage large-scale genomic datasets, facilitating the identification of differentially expressed genes and their correlations with clinical outcomes. The primary objective of this research is to elucidate the relationship between CPLX1 expression levels and key clinical features, as well as to explore its potential implications in the context of immune cell infiltration and ferroptosis pathways. By employing advanced statistical analyses, including Kaplan-Meier survival plots and Cox regression models, this study aims to provide insights into the prognostic significance of CPLX1 in CRC, thereby contributing to the understanding of its role in cancer biology and treatment resistance.

Here, our study underscores the pivotal role of CPLX1 as a prognostic biomarker in colorectal cancer, revealing its significant association with immunotherapy resistance and ferroptosis. The correlation between elevated CPLX1 expression and adverse clinical outcomes highlights its potential as a therapeutic target, offering a promising avenue for enhancing treatment efficacy in CRC.

## 2 Materials and methods

### 2.1 Datasets

RNA-seq data from the TCGA database (https://portal.gdc.cancer.gov) were downloaded and collated for the TCGA-COAD and TCGA-READ Project STAR processes and extracted in TPM format. Differentially expressed genes were also evaluated in the GEO (http://www.ncbi.nlm.nih.gov/geo/) GSE156451 dataset in 47 patients with primary colon tumor and 48 normal colons.

### 2.2 TIMER 2.0

The TIMER 2.0 database allows the use of TCGA data to investigate differential expression of genes of interest between tumor and control tissues (8). Thus, TIMER 2.0 was used to determine CPLX1 levels in various cancers.

### 2.3 Survival analysis

Kaplan-Meier plots, together with log-rank assessments, probed survival, utilizing the median level of CPLX1 expression as a cut-off value. The links across clinical features and survival were assessed through univariate/multivariate Cox regression with major parameters (P<0.05) from the univariate analysis employed within the multivariate analysis. Forest maps were constructed using the R package “ggplot2”.

### 2.4 Mutational analysis of CPLX1 in COAD

The frequency of CPLX1 mutations in COAD was assessed using cBioPortal, and specific types of mutations were analyzed using the COSMIC database.

### 2.5 Identification of differentially/co-expressed genes

Genes co-expressed with CPLX1 in CRC were investigated using LinkedOmics (9) and TCGA-derived RNA-seq datasets. Pearson’s correlation coefficient was used to explore co-expression, and volcano plots were generated from the LinkedOmics website. Heatmaps were created in R software (version 3.6.3) using limma. Patients in the TCGA dataset were assigned to the CPLX1 low and CPLX1 high groups based on median CPLX1 transcriptome levels, and differentially expressed genes (DEGs) were identified and plotted for all cohorts using the limma package. Venn diagrams were plotted to identify overlap between DEGs and co-expressed genes.

### 2.6 Functional enrichment analysis

The functions of the identified overlapping genes were explored by GO and KEGG analysis in metscape (10).

### 2.7 GSEA analysis

We employed the GSEA computational method to assess the statistical significance of a preselected gene set. Following correlation analysis, we generated an initial list of gene categories. These categories were then segmented into various groups for each analysis, involving, 1000 permutations of gene sets to identify any disparities among them. The results of this analysis aided in identifying the critical genetic functions and signaling pathways linked to CPLX1.

### 2.8 Immune infiltration

Single-sample GSEA (ssGSEA) analysis was performed to detect the relationship between immune cell infiltration and gene expression in hepatocellular carcinoma tumors by means of the R-package GSVA package. Gene expression profiles of 24 immune cell types were studied. The association between CPLX1 levels and infiltrating cell subpopulations was tested by the Spearman and Wilcoxon rank sum test.

### 2.9 Relationships between expression of CPLX1 and ferroptosis-associated genes

Potential relationships between the expression of CPLX1 and that of ferroptosis-associated genes were evaluated in TCGA-COAD/READ datasets using R, which was also used to assess the proportions of ferroptosis-associated genes in samples with high or low CPLX1 levels. The “box plot” package in R was used to visualize the results.

### 2.10 Cell culture and experiments

The RKO and HCT8 cell line was acquired from Servicebio Biotechnology Co. (Wuhan, China). The cells were maintained in DMEM with 10% fetal bovine serum and 10 µL/mL penicillin/streptomycin at 37°C and 5% CO_2_ with saturated humidity.

### 2.11 Transfection with small interfering RNA

RKO and HCT8 cells (2×10^4^/well) were inoculated in 48-well plates and transfected with CPLX1 siRNA or siRNA negative control, as described in a manual (GenePharma, Shanghai, China). The siRNAs were mixed with 2.5 µL/100 µL of Lipofectamine 3,000 (Invitrogen, MA, USA) in a serum-free medium and allowed to stand for 20 min, after which the mixture was added to 250 µL medium containing 10% FBS. Lastly, 250 µL of this solution was placed in each well and incubated for 38 h under standard conditions. The CPLX1 siRNA sequences were for siCPLX1 #1: forward: 5’-GAGGAGAAGGAGAAGAAGAAG-3’ and reverse: 5’-CUUCUUUCUUCUUUCUUCUUdTdT-3’, and #2: forward: 5’-AACTGCTGAGGTCGAGGAGA-3’ and reverse: 5’-TTCAGCAGTCACGTCCTCT-3’. The control (si-Control) sequences were forward: 5’-UUCUCCGAACGUGUCACGUTT-3’ and reverse: 5’-ACGUGACACGUUCGGAGAATT-3’. The siRNAs were synthesized by GenePharma Co., Ltd. (Shanghai, China).

### 2.12 The quantitative real-time polymerase chain reaction

Total cellular RNA was extracted using Trizol reagent (Solarbio Biotechnology Co., Ltd., Beijing, China). Log-phase cells were taken. One milliliter of Trizol reagent was added to the cells, blown up with a pipette and ice-bath for 5 minutes to completely separate the nucleoprotein complexes. The content and purity of RNA were determined using a NanoDrop spectrophotometer (Thermo Fisher Scientific, USA). The purity of RNA was considered satisfactory when the OD_260 nm_/OD_280 nm_ values were between 1.8 and 2.0. The quantitative real-time polymerase chain reaction (qRT-PCR) was performed in two steps. The reaction conditions were as follows: pre-denaturation at 95.0 °C for 30 s, denaturation at 95.0 °C for 10 s, annealing and extension at 60.0 °C for 30 s, and 40 cycles. The quantitative real-time PCR primers for CPLX1 and glyceraldehyde-3-phosphate dehydrogenase (GAPDH) were as follows: forward: 5’-ACGCCGCCAAGAAGGAGAGAG-3’, reverse: 5’-GATGCCGTACTTGTCTCGGATGC-3’; forward: 5’-CAGGAGGCATTGCTGATGAT-3’, reverse: 5’-GAAGGCTGGGGCTCATTT-3’. The relative expression of the target genes in each group was analyzed by the 2^-ΔΔCt^ method, where ΔΔCt = ΔCt (experimental group) - ΔCt (control group), ΔCt = Ct (target gene) - ΔCt (GAPDH), and Ct is the number of amplification cycles required for the fluorescence intensity to reach the threshold value.

### 2.13 Western blotting

RKO and HCT8 cells were lysed in RIPA buffer (P0013B, Beyotime Biotechnology, Shanghai, China) containing PMSF (ST506, Beyotime Biotechnology) and phosphatase inhibitor (PPI, P1081, Beyotime Biotechnology). After separation on SDS-PAGE, the proteins were electroblotted onto PVDF membranes (0.45 µm, Merck Millipore Ltd., Darmstadt, Germany) and blocked for 1 h with 5% BSA. The blots were then treated with primary antibodies, rabbit anti-CPLX1 (10246-2-AP, Proteintech, Wuhan, China; 1:2000) and rabbit anti-GAPDH (10494-1-AP, Proteintech, Wuhan, China; 1:10000), followed by secondary antibodies. The bands were analyzed using a gel-imaging system (Tanon-4600, Tanon, Shanghai, China).

### 2.14 Cell proliferation and viability assay

Transfected RKO and HCT8 cells (5,000/well) were grown in 96-well plates. After 24 h, 10 µL of CCK-8 solution (MCE, USA) was added, and absorbances at 450 nm were read after 2 h using a microplate reader (Thermo Fisher Scientific, Waltham, MA). Cell viability is represented by optical density (OD) values. Continuous testing for 5 days.

### 2.15 Colony formation assay

Different types of cells in the logarithmic growth phase counted, cells seeded at 500 pcs/well in 6-well plates, incubated with cells for one week. Observe the formation of punctate clones at the bottom of the 6-well plates, fix cells with 4% tissue fixative solution (Solarbio Biotechnology Co., Ltd., Beijing, China), stain 1% crystal violet for 30 min, and take pictures for analysis.

### 2.16 Wound-healing assays

Transfected RKO and HCT8 cells were grown in 6-well plates until confluent. After removing detached cells by rinsing with PBS, scratches were made in the cell monolayer with a 200µL pipette tip. The cells were kept in a serum-free medium and imaged after 0, 24, and 48 h. Data were analyzed using Image J 2.3.0. Cell wound healing rate = (Scratch width_0h_ – Scratch width_24h_)/Scratch width_0h_ × 100%.

### 2.17 Transwell assay

Suspend cells with serum-free medium, regulate the concentration of cells at 5 × 10^4^ pcs/well, seed in the upper chamber of the Transwell chambers pre-lined with matrix gel, purchased from Corning (#354480, Corning^®^ BioCoat™ Matrigel^®^ Coated Invasion Chambers), and spread 20% of the medium in the lower chamber. Incubate in 37°C 5%CO_2_ incubator for 24–48 hours. Fix the upper chamber with 4% tissue fixative, stain with crystal violet for 30 min, wipe the inside of the upper chamber with a cotton swab, observe under a microscope and take pictures, count for analysis.

### 2.18 Detection of intracellular MDA content

The detection of malondialdehyde (MDA) content was carried out by the colorimetric method of thiobarbituric acid (TBA) (S0131S, Beyotime Biotechnology Co.,Ltd). First, cell or tissue samples were collected, lysed or homogenized, and the supernatant was taken as the sample to be tested. Subsequently, the samples were mixed with 10% trichloroacetic acid (TCA), centrifuged to remove proteins, and then the supernatant was taken to react with TBA reagent and heated in a water bath at 95°C for 15 min, so as to generate a red product between MDA and TBA. After cooling, the absorbance value was measured at 532 nm, and the content of MDA in the sample was calculated by standard curve.

### 2.19 Measurement of intracellular Fe^2+^ content

In this study, Fe²^+^ content was determined by o-phenanthroline colorimetric method (BC5410, Beijing Solarbio Science & Technology Co., Ltd). First, an appropriate amount of cell or tissue sample was taken and after lysis or homogenization, the supernatant was taken as the sample to be tested. Subsequently, a reaction reagent containing o-phenanthroline and ferrous sulfate was added to the sample, and Fe²^+^ combined with o-phenanthroline to form a red complex. The absorbance value was measured at 510 nm and the amount of Fe²^+^ in the sample was calculated by standard curve method.

### 2.20 Lactate dehydrogenase assay to detect cell death

In this study, we used the Lactate Dehydrogenase (LDH) Assay Kit (40209ES76, YEASEN Biotech Co., Ltd) to assay the release of LDH from cell culture supernatants to assess the degree of cell injury. An appropriate amount of cells was inoculated into 96-well cell culture plates according to the size and growth rate of the cells so that the cell density did not exceed 80-90% when the cells were to be assayed after 48 hours. After reaching the predetermined time, the cell culture plate was centrifuged in a multiwell plate centrifuge at 400 g for 5 min. 120 μL of supernatant from each well was taken separately and added to the corresponding well of a new 96-well plate, followed by sample determination on an enzyme labeling instrument (490 nm). The unit of LDH activity in the sample to be measured (mU/mL) = (OD_490_ of sample wells - OD_490_ of background blank control wells)/(OD_490_ of standard tubes - OD_490_ of standard blank tubes) × concentration of standard (mU/mL).

### 2.21 Experiments on subcutaneous tumor formation in nude mice

In this study, nude mice subcutaneous tumor formation assay was used to assess the tumorigenicity of CPLX1 for RKO cells. Female BalB/C nude mice aged 6–8 weeks were used for the experiments. Cells were collected from the logarithmic growth phase, adjusted to the appropriate concentration (1 × 10^6^ cells/100 µL), mixed with matrix gel (Matrigel) at a 1:1 volume ratio, and then inoculated into the subcutaneous tissues in the axillae of both sides of the nude mice. Each nude mouse was inoculated with one locus, and six nude mice were set up in each group to ensure the reproducibility of the experiment. After inoculation, the nude mice were kept in an SPF-grade environment, and were regularly observed and tumor volume was measured using vernier calipers (volume = length × width²/2).

### 2.22 Immunohistochemical assays

Immunohistochemical assays were performed using routine steps. First, the tissue samples were fixed in 4% paraformaldehyde solution, followed by dehydration, transparency, paraffin embedding, and cutting into sections of 4 μm thickness. After the sections were dewaxed to water, they were processed for antigenic repair and thermally repaired using citrate buffer (pH 6.0). Subsequently, sections were treated with 3% hydrogen peroxide solution to block endogenous peroxidase activity, followed by closure of non-specific binding sites with normal goat serum. After that, the sections were incubated with primary antibody (anti-CPLX1, 10246-2-AP, Proteintech, Wuhan, China, 1:100 dilution; anti-Ki67, 27309-1-AP, Proteintech, Wuhan, China, 1:4000 dilution; anti-E-cadherin, 20874-1-AP, Proteintech, Wuhan, China, 1:5000 dilution; anti-N-cadherin, 22018-1-AP, Proteintech, Wuhan, China, 1:8000 dilution) at 4°C overnight. On the following day, the sections were washed with PBS, and then biotin labeled goat anti-mouse secondary antibody working solution (SAP-9100, Beijing Zhongshan Golden Bridge Biotechnology Co., Ltd) were added and incubated for 1 hour at room temperature. Finally, the sections were color developed by DAB, re-stained by hematoxylin, dehydrated, transparent and then sealed, observed by light microscope and photographed to record the results.

### 2.23 Resolving changes and functions of CPLX1 at the single-cell level

Download Colorectal cancer single-cell sequencing dataset GSE166555 for in-depth analysis of CPLX1 changes and function. Bioinformatics tools were used to process and analyze the massive single-cell transcriptome data. Different cellular subpopulations were identified by clustering analysis (e.g., t-SNE or UMAP downscaling), and characterized genes of each subpopulation were screened in combination with differential expression analysis (e.g., DESeq2 or Wilcoxon rank sum test). In addition, we applied cell type annotation tools (e.g. SingleR or CellMarker) to functionally annotate cell subpopulations.

### 2.24 Statistical analysis

Bioinformatics data were assessed through R software (Version 3.6.3, R Foundation for Statistical Computing, Vienna, Austria). Differences were evaluated using *t*-tests and two-way ANOVA for separate specimens. *P*<0.05 was deemed to confer statistical significance. Relationships between CPLX1 levels and patient clinicopathological features were analyzed by χ^2^ assessments, logistic regression, and Fisher’s exact and Wilcoxon rank-sum assessments.

## 3 Results

### 3.1 Patients’ baseline features

Data on 644 CRC patients were retrieved from TCGA in November 2024 and are shown in **Table 1**. Patients were allocated to two groups (n=322 and n=322, respectively) based on their CPLX1 levels. The relationships between the CPLX1 levels and clinicopathological features were then analyzed, finding that the CPLX1 levels were significantly linked to pathologic T stage and lymphatic invasion (all *P*<0.05).

**Table 1:** Clinicopathological features for CRC patients in relation to CPLX1 level.

Characteristics	Low expression of CPLX1	High expression of CPLX1	*P* value
n	322	322	
Pathologic T stage, n (%)			< 0.001
T1&T2	85 (13.3%)	46 (7.2%)	
T3&T4	235 (36.7%)	275 (42.9%)	
Pathologic N stage, n (%)			0.744
N0	188 (29.4%)	180 (28.1%)	
N1	76 (11.9%)	77 (12%)	
N2	56 (8.8%)	63 (9.8%)	
Pathologic M stage, n (%)			0.187
M0	239 (42.4%)	236 (41.8%)	
M1	38 (6.7%)	51 (9%)	
Pathologic stage, n (%)			0.265
Stage I & Stage II	180 (28.9%)	169 (27.1%)	
Stage III & Stage IV	129 (20.7%)	145 (23.3%)	
Primary therapy outcome, n (%)			0.852
PD&SD	21 (6.7%)	17 (5.4%)	
PR&CR	147 (47.1%)	127 (40.7%)	
Gender, n (%)			0.179
Female	142 (22%)	159 (24.7%)	
Male	180 (28%)	163 (25.3%)	
Race, n (%)			0.831
Asian & Black or African American	43 (10.9%)	38 (9.6%)	
White	162 (41.1%)	151 (38.3%)	
Age, n (%)			0.750
<= 65	140 (21.7%)	136 (21.1%)	
> 65	182 (28.3%)	186 (28.9%)	
CEA level, n (%)			0.136
<= 5	135 (32.5%)	126 (30.4%)	
> 5	68 (16.4%)	86 (20.7%)	
Perineural invasion, n (%)			0.080
No	87 (37%)	88 (37.4%)	
Yes	22 (9.4%)	38 (16.2%)	
Lymphatic invasion, n (%)			0.019
No	187 (32.1%)	163 (28%)	
Yes	101 (17.4%)	131 (22.5%)	

### 3.2 CPLX1 levels are significantly raised in CRC

**Figure 1:**
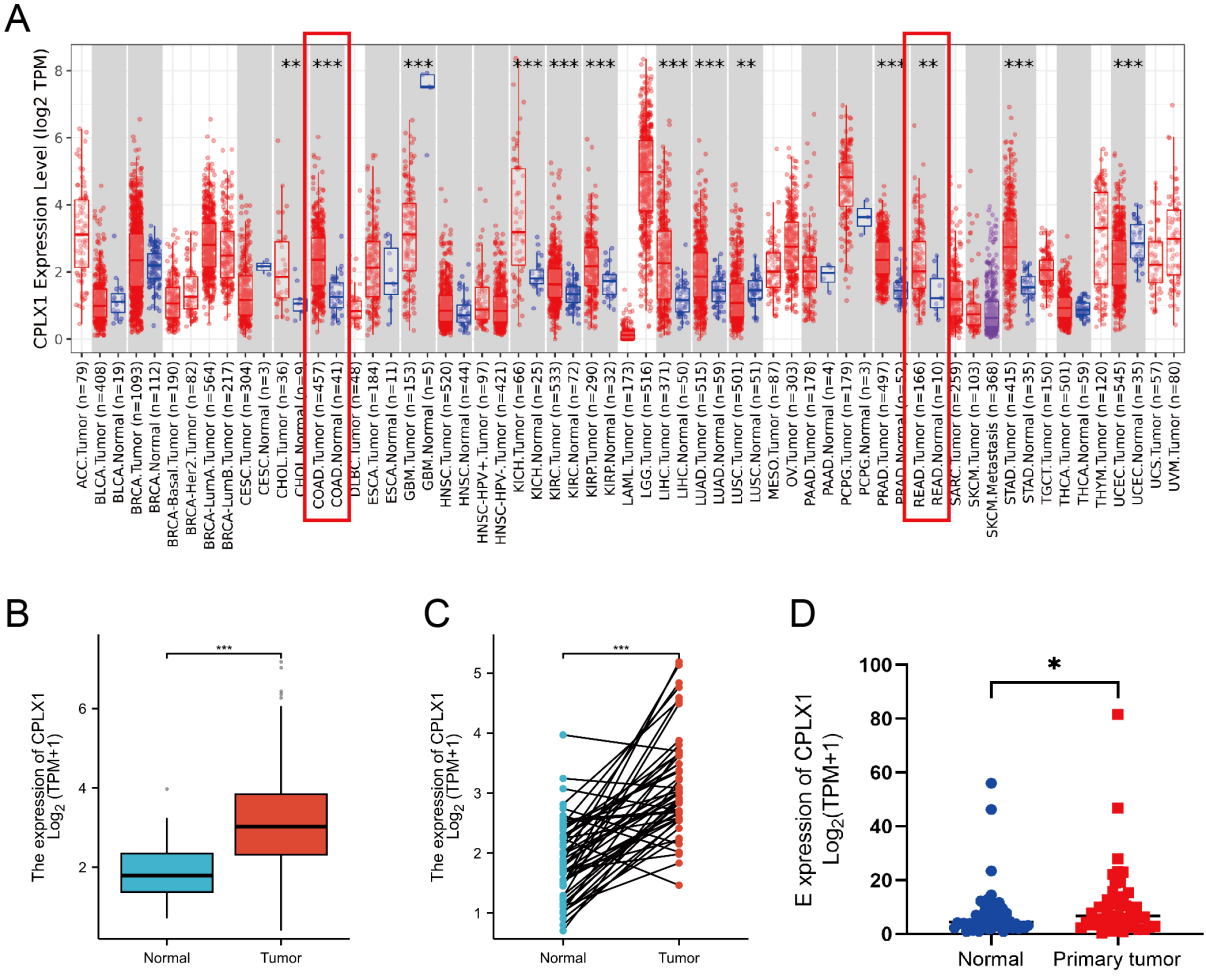
CPLX1 expression profile across differing cancer models. **(A)** CPLX1 levels within various tumors, determined through TIMER2.0; **(B)** CPLX1 levels across tumor/healthy specimens from TCGA; **(C)** CPLX1 levels in paired tumor and normal samples (TCGA); **(D)** CPLX1 mRNA levels in CRC vs. controls from the GSE156451 dataset. TCGA, The Cancer Genome Atlas. CRC, colorectal cancer. *P<0.05; **P<0.01; ***P<0.001.

The first step undertaken was surveying the levels of CPLX1 in tumor and control samples using TIMER2.0. This showed significantly higher levels of CPLX1 in tumor tissues relative to the controls (**Figure 1A**). CPLX1 showed raised expression in other types of cancer, including kidney renal clear cell carcinoma, liver hepatocellular carcinoma and lung adenocarcinoma. Reduced levels of CPLX1 were, however, observed in lung squamous cell carcinoma and uterine corpus endometrial carcinoma. The relationships between CPLX1 levels and clinical features were evaluated using TCGA data. This indicated that, in agreement with the above findings, CPLX1 levels were markedly raised in the CRC tissues compared to the controls (*P*<0.001; **Figure 1B**). A further comparison of paired CRC and normal tissues confirmed these findings, showing elevation of CPLX1 in tumor tissues (*P*<0.001; **Figure 1C**). A GEO database analysis confirmed these results (*P*<0.05; **Figure 1D**).

### 3.3 CPLX1 is associated with CRC clinical features

**Figure 2:**
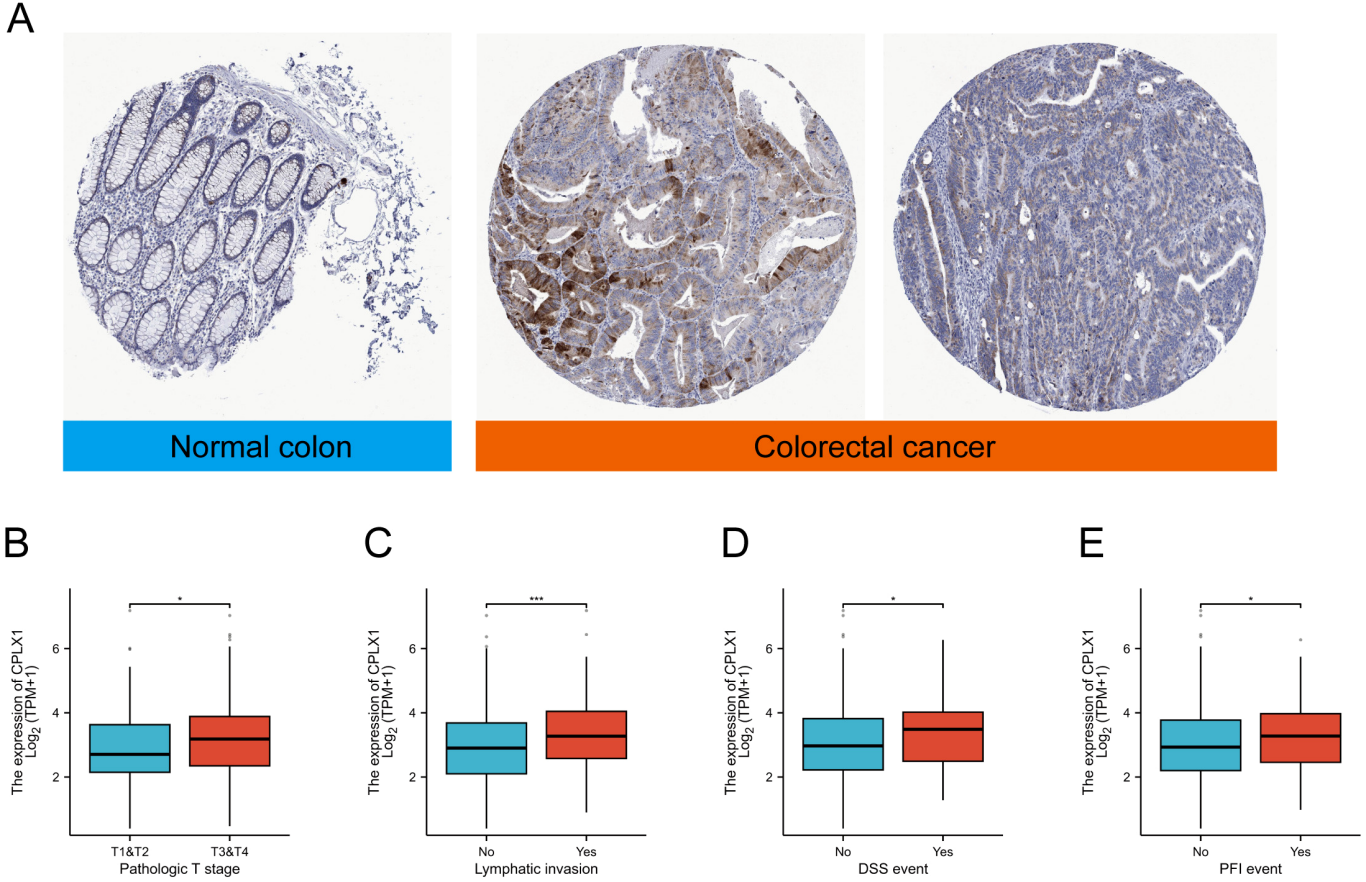
CPLX1 mRNA levels in relation to clinical features. **(A)** IHC of CPLX1 in CRC tissue and normal colon; **(B)** Relationship between CPLX1 mRNA levels and T stage; **(C)** Links across CPLX1 mRNA expression and lymphatic invasion; **(D)** Links across CPLX1 mRNA levels and DSS event; **(E)** Relationship between CPLX1 mRNA levels and PFI event. CRC, colorectal cancer. *P<0.05; ***P<0.001.

IHC was then used to examine CPLX1 in CRC samples. CPLX1 was found to be upregulated in colorectal cancer material compared with the normal colon samples (**Figure 2A**). This implies that CPLX1 modulates CRC development and progression. Clinical information and CPLX1 levels in 644 patients with CRC were obtained from the TCGA. Associations between these parameters were examined by univariate analysis, finding a significant association between elevated CPLX1 levels and pathologic T stage and lymphatic invasion (**Figures 2B, C**, **Table 2**). The dataset outcomes suggested CRC cases with upregulated CPLX1 had raised odds of experiencing advanced disease in comparison with those with lower CPLX1 levels (**Figures 2D, E**).

**Table 2:** Links across CPLX1 levels and CRC clinicopathological characteristics, shown through logistic regression assessment.

Characteristics	Total (N)	OR (95% CI)	*P* value
Pathologic T stage (T3&T4 vs. T1&T2)	641	2.162 (1.451 – 3.222)	**< 0.001**
Pathologic N stage (N1&N2 vs. N0)	640	1.108 (0.810 – 1.516)	0.522
Pathologic M stage (M1 vs. M0)	564	1.359 (0.861 – 2.146)	0.188
Pathologic stage (Stage III & Stage IV vs. Stage I & Stage II)	623	1.197 (0.872 – 1.643)	0.265
Primary therapy outcome (PR&CR vs. PD&SD)	312	1.067 (0.540 – 2.111)	0.852
Gender (Male vs. Female)	644	0.809 (0.593 – 1.103)	0.180
Race (White vs. Asian & Black or African American)	394	1.055 (0.647 – 1.721)	0.831
Age (> 65 vs. <= 65)	644	1.052 (0.770 – 1.437)	0.750
CEA level (> 5 vs. <= 5)	415	1.355 (0.908 – 2.022)	0.137
Perineural invasion (Yes vs. No)	235	1.708 (0.934 – 3.121)	0.082
Lymphatic invasion (Yes vs. No)	582	1.488 (1.066 – 2.078)	**0.020**

Bold shows *p*<0.05.

### 3.4 CPLX1 as a predictor of CRC prognosis

**Figure 3:**
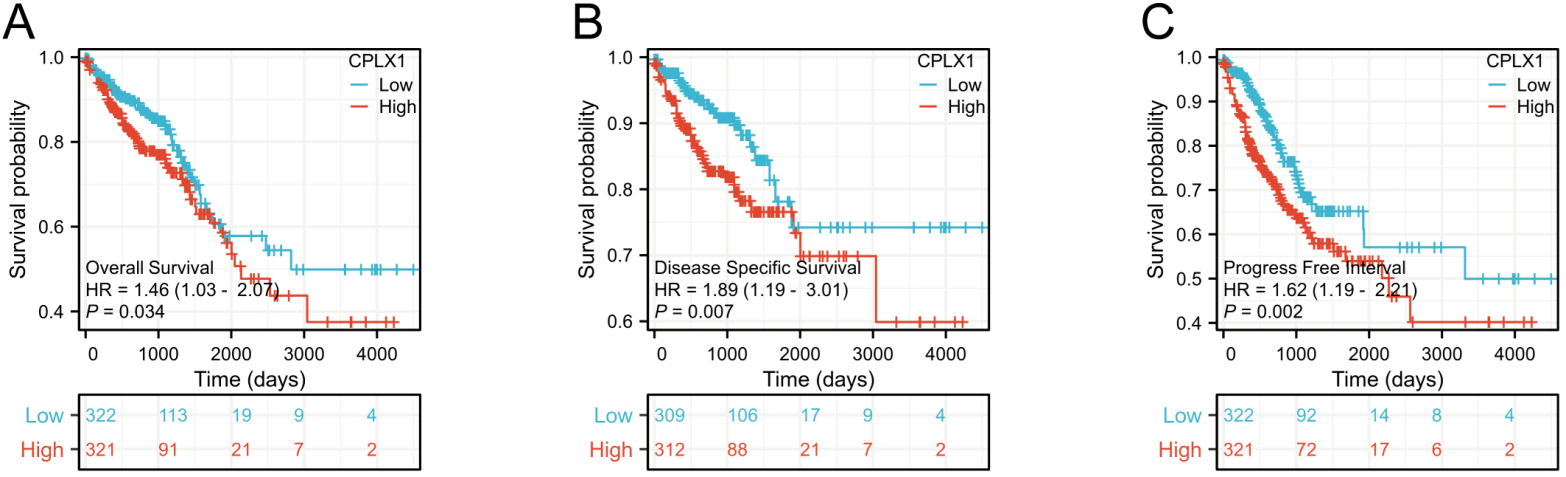
Prediction of prognosis by CPLX1 in patients with CRC. Kaplan-Meier plots showing OS **(A)**, DSS **(B)**, and PFI **(C)** compared to CPLX1 levels (*P*<0.05). Red curves represent high CPLX1 expression, while blue indicates low CPLX1 expression. CRC, colorectal cancer; OS, overall survival; DSS, disease-specific survival; PFI, progression-free interval.

Kaplan-Meier survival plots demonstrated that cases having upregulated CPLX1 had significantly reduced OS (HR=1.46, *P*=0.034) (**Figure 3A**), disease-specific survival (DSS) (HR=1.89, *P*=0.007) (**Figure 3B**), together with progression-free intervals (PFI) (HR=1.62, *P*=0.002) (**Figure 3C**) in comparison to cases having downregulated CPLX1. Furthermore, multivariate regression analysis showed that CPLX1 provided separate predictions for OS (HR=1.460, *P*<0.01), DSS (HR=1.895, *P*<0.01) and PFI (HR=1.623, *P*<0.01) (**Table 3**).

**Table 3:** Survival outcomes of patients with CRC in relation to clinicopathological features and CPLX1, shown by regression analysis.

Characteristics	HR for OS (95% CI)		HR for DSS (95% CI)		HR for PFI (95% CI)	
	Univariate analysis	Multivariate analysis	Univariate analysis	Multivariate analysis	Univariate analysis	Multivariate analysis
**T stage (T1&T2 vs. T3&T4)**	2.468	10.125	6.440	70462653.4804	3.198	1.529
**M stage (M0 vs. M1)**	3.989	0.445	7.471	0.000	5.577	0.879
**Pathologic stage (Stage I & Stage II vs. Stage III & Stage IV)**	2.988	220.407	5.716	–	2.924	27.842
**Primary therapy outcome (PD&SD vs. PR&CR)**	0.109	0.173	0.055	0.212	0.097	0.138
**Age (<=65 vs. >65)**	1.939	1.372	1.421	–	1.006	–
**Lymphatic invasion (No vs. Yes)**	2.144	2.143	3.669	3.763	2.358	1.399
**CPLX1 (Low vs. High)**	1.460	1.460	1.895	1.895	1.623	1.623

### 3.5 Associations between CPLX1 and prognosis in CRC patient subgroups

**Figure 4:**
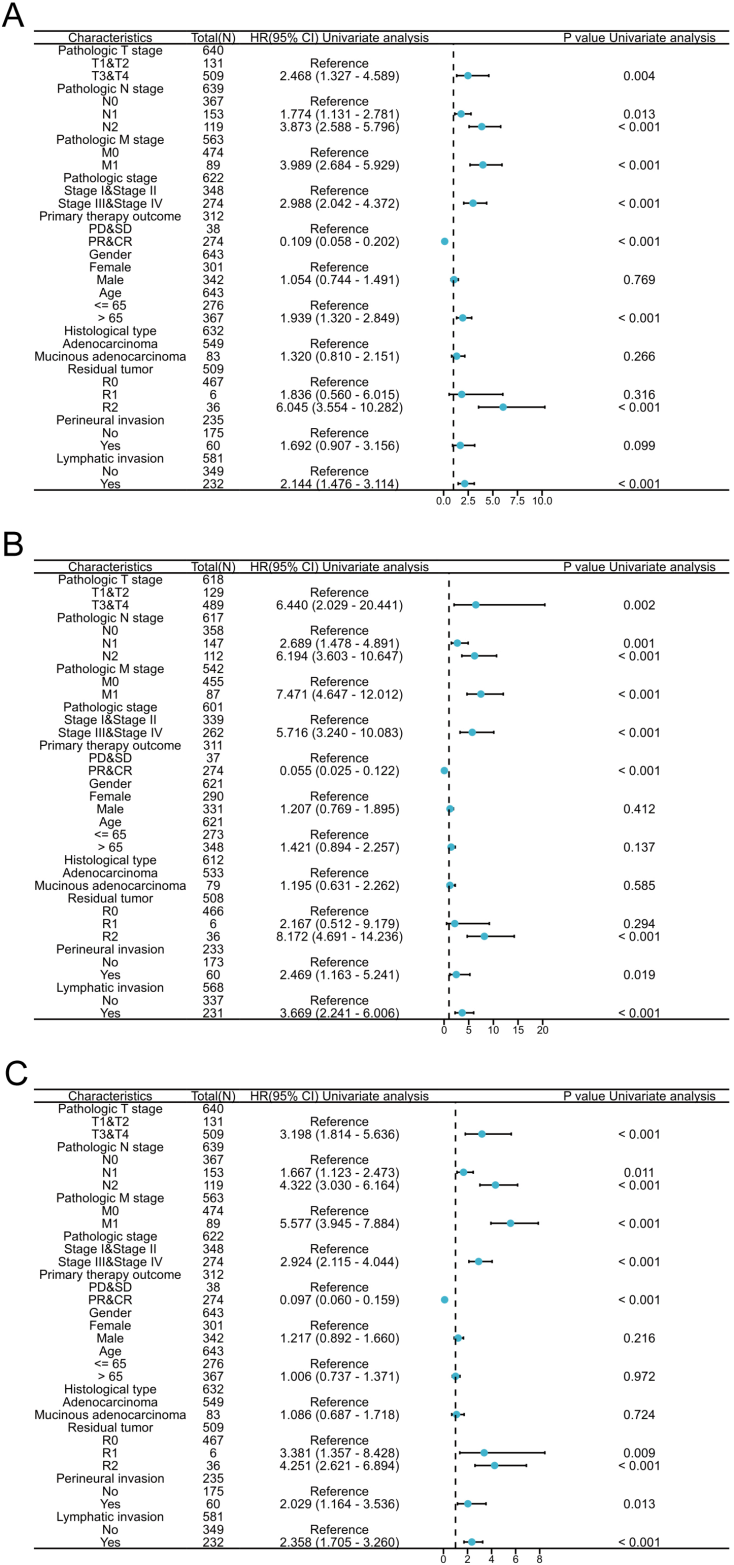
CPLX1 level is a prognostic predictor in various patient subgroups. Regression results for OS **(A)**, DSS **(B)**, and PFI **(C)** according to subgroups. Results represent HRs, bars indicate 95% CIs, and diamond sizes indicate the degree of CPLX1 prognostic ability. OS, overall survival; DSS, disease-specific survival; PFI, progress-free interval; HR, hazard ratio; 95% CI, 95% confidence interval.

The predictive ability of CPLX1 in relation to clinicopathological features was analyzed using Cox regression (**Figure 4**). High CPLX1 levels were linked to lower OS in patients of various ethnicities, in particular, T and M pathologic stage (HR=2.468, *P*=0.004; HR=3.989, *P*<0.001), primary therapy outcome (HR=0.109, *P*<0.001), age (HR=1.939, *P*<0.001) and lymphatic invasion (HR=2.144, *P*<0.001) (**Figure 4A**), DSS (**Figure 4B**), and PFI (**Figure 4C**). This indicates an association between increased CPLX1 levels and shorter survival in CRC patients.

### 3.6 CPLX1 is an effective biomarker for CRC diagnosis

**Figure 5:**
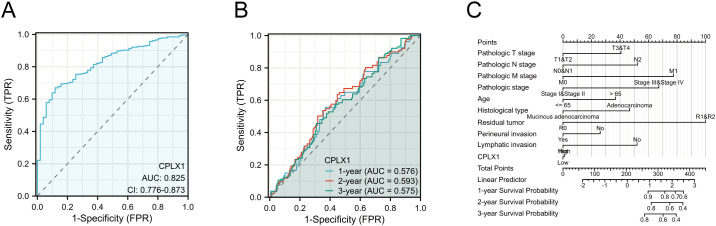
Performance of the nomogram, including CPLX1, for predicting CRC diagnosis and outcomes. **(A)** ROC curve for CPLX1 differentiation between tumor and control tissues; **(B)** Time-dependent ROC curves for predicting 1-, 2-, and 3-year survival based on CPLX1 levels; **(C)** Efficacy of the nomogram, which includes various clinicopathological parameters and CPLX1, for predicting 1-, 2-, and 3-year survival outcomes. CRC, colorectal cancer; TPR, true positive rate; FPR, false positive rate; ROC, receiver operating characteristic curve.

ROC curves showed an AUC of 0.825 for CPLX1, indicating that it accurately distinguishes between tumor and normal control samples (**Figure 5A**). Time-dependent analysis of the ROC curves indicated AUC values above 0.5 for the prediction of 1-, 2-, and 3-year survival by CPLX1 (**Figure 5B**). We then constructed a nomogram based on CPLX1 level, which were identified by multivariate analysis as being significantly associated with patient prognosis. The nomogram was found to effectively predict the 1-, 2-, and 3-year likelihood of survival in CRC patients (**Figure 5C**).

### 3.7 Genetic changes in CPLX1 are not linked to survival

**Figure 6:**
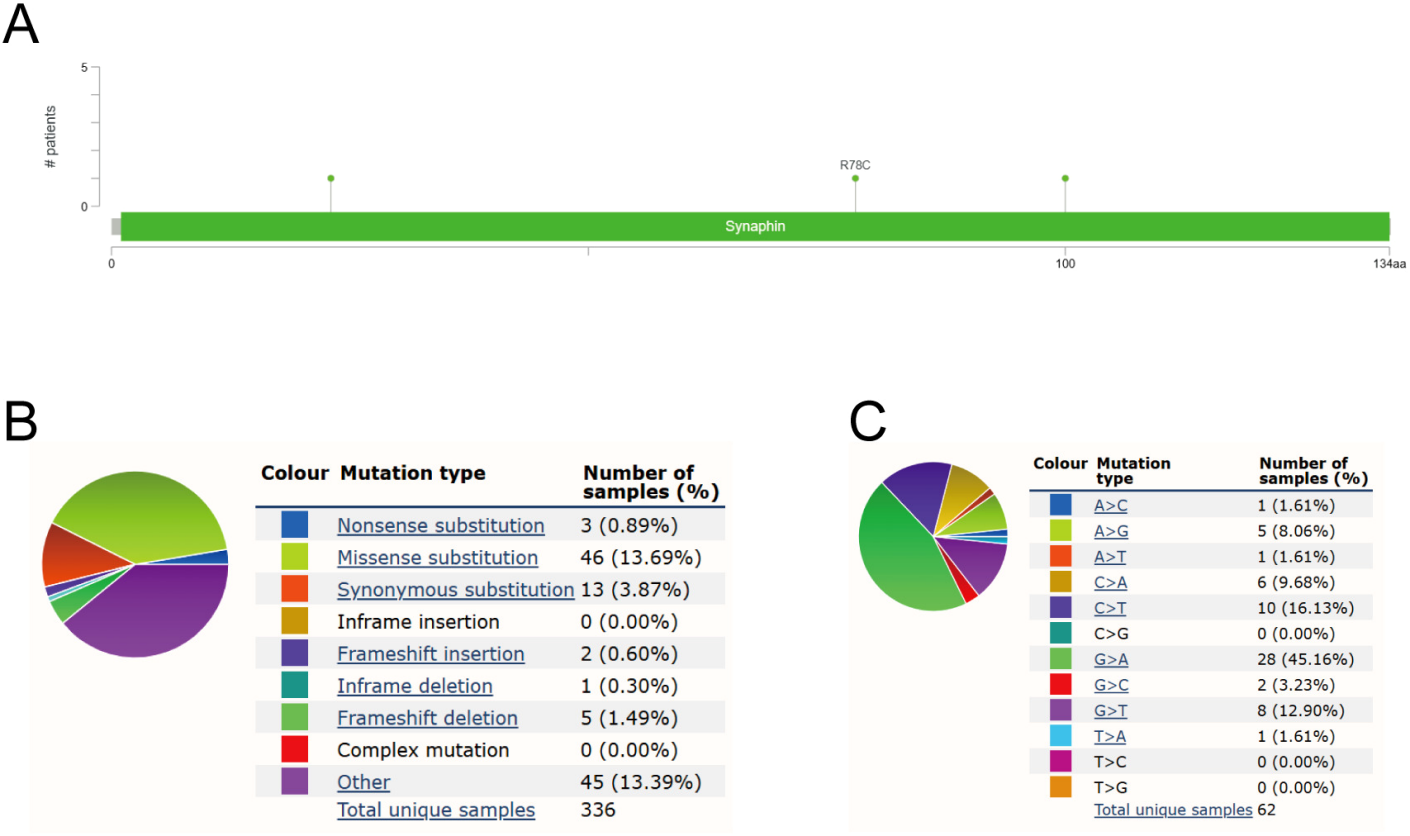
CPLX1 mutations in CRC. **(A)** Schematic showing CPLX1 mutations in CRC, determined by cBioPortal; **(B)** Types of CPLX1 mutations identified using COSMIC database; **(C)** Alteration frequency of CPLX1 identified by COSMIC database. CRC, colorectal cancer.

A growing number of studies have demonstrated that oncogene mutations have an important regulatory role in ferroptosis and tumor progression, which inhibit the onset of ferroptosis and promote tumor proliferation, migration, and invasion, and even affect the sensitivity of tumor cells to therapy (11–14). Therefore, we wanted to analyze the mutation of CPLX1 in colorectal cancer. Mutation frequencies in CPLX1 were assessed using the cBioPortal online resource. All five datasets, namely, AMC, MSK, RIKEN, INSERM, and TCGA Pan-Cancer Atlas, were analyzed, comprising 1,000 samples. The overall frequency of somatic mutations in CPLX1 associated with CRC was found to be 0.03%, representing a low figure of just three mutations per 1,0000 samples, most of which were missense mutations (**Figure 6A**). This indicated an absence of an association between CPLX1 mutations and CRC patient prognosis. The types of CPLX1 mutations were also assessed using the COSMIC database. **Figure 6** shows two pie charts illustrating the different types of mutation. Approximately 13.69% of samples contained missense mutations, with synonymous mutations in 3.87% (**Figure 6B**). Most substitutions involved G>A (45.16%), with C>T accounting for 16.13% and G>T, 12.90% (**Figure 6C**).

### 3.8 Functional annotation and pathway enrichment of CPLX1-associated genes within CRC

**Figure 7:**
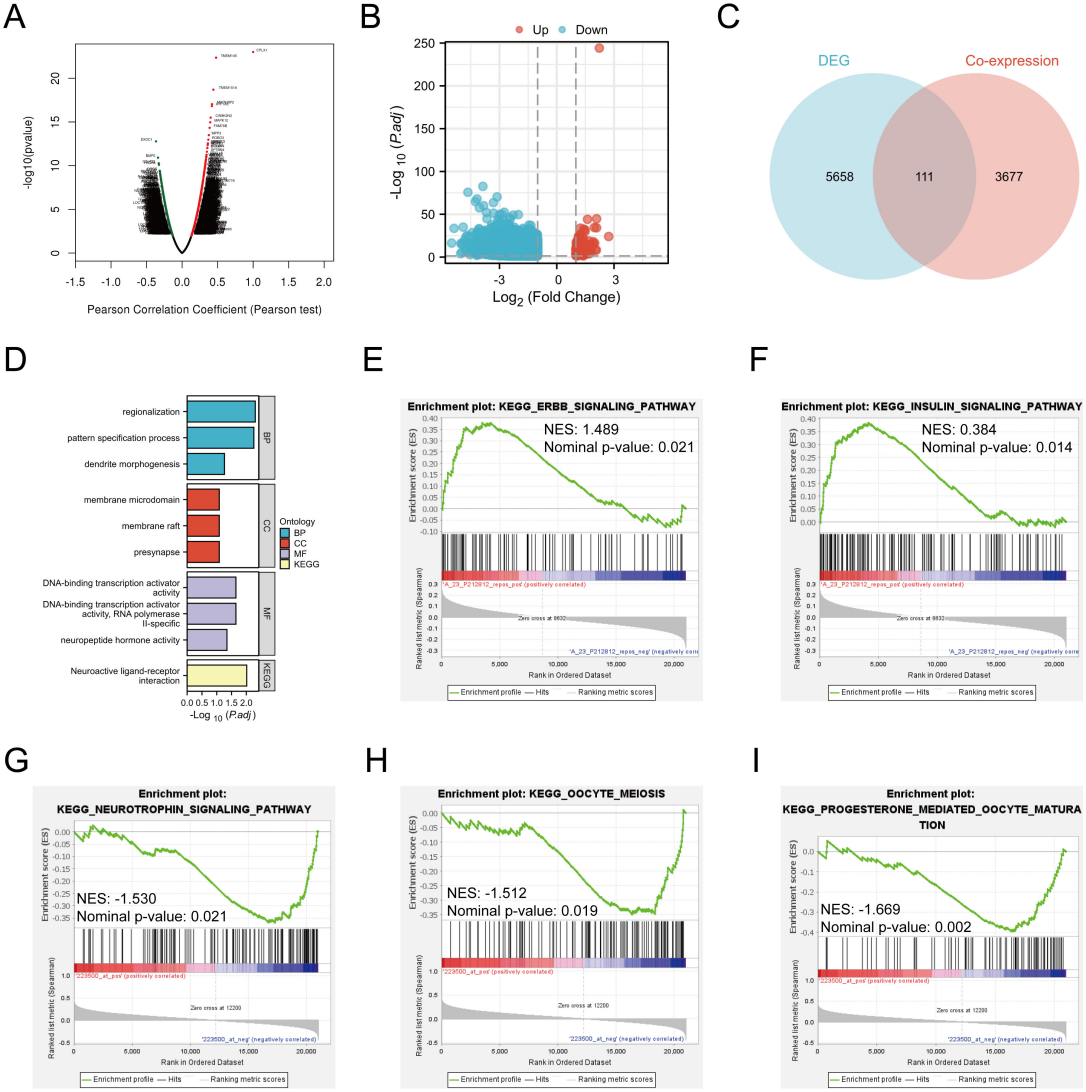
CPLX1 associations with other genes in CRC. **(A)** Volcano plot showing co-expression of genes with CPLX1, compiled by LinkedOmics; **(B)** Volcano plot of CPLX1^high^ and CPLX1^low^ differential genes; **(C)** Venn diagram showing overlap across CPLX1-co expressed genes and DEGs; **(D)** Pathways showing enrichment of 111 overlapping genes, as indicated by GO and KEGG assessments. CRC, colorectal cancer; DEG, differentially expressed gene; BP, biological process; CC, cellular component; MF, molecular function; KEGG, Kyoto Encyclopedia of Genes and Genomes; **(E-I)** GSEA enrichment analysis of CPLX1.

To probe possible functions for CPLX1 within CRC, genes found to be co-expressed with CPLX1 in TCGA were further explored using LinkedOmics (**Figure 7A**). This showed significant correlations between CPLX1 and 3,788 genes that showed co-expression in CRC [false discovery rate (FDR)<0.05, *P*<0.05]. Of these genes, 2,155 were found to be positively associated with the expression of CPLX1, while negative correlations were seen for 1,633 genes. The identified DEGs between the CPLX1^high^ and CPLX1^low^ groups in CRC were then investigated. In all, 5,960 DEGs were observed (*P*<0.05, |log_2_ FC|>=1) (**Figure 7B**), of which 163 were upregulated and 5,797 of which were downregulated in the CPLX1^high^ group. Venn diagrams, compiled by the Draw Venn Diagrams online tool, were used to compare the overlap between the DEGs and the genes found to be co-expressed with CPLX1 when comparing these DEGs of CPLX1.The diagrams showed that 111 genes overlapped between the two populations (**Figure 7C**). Of these genes, 111 were then subjected to functional analysis to clarify the possible role of CPLX1. GO and KEGG enrichment analyses indicated that “regionalization” and “membrane microdomain” were likely linked to the function of CPLX1 in CRC pathogenesis. GO annotation and enrichment suggested most genes were enriched within pathways related to activities of ion channels and the biology of extracellular membranes, including DNA-binding transcription activator activity, pattern specification process and Neuroactive ligand-receptor interaction, among others (**Figure 7D**). Then, in order to further analyze the molecular mechanism of CPLX1 in promoting colorectal cancer development, we selected two colorectal cancer datasets, GSE17538 and GSE8671, and the “c2.cp.kegg_legacy.v2024.1.Hs.symbols” panel for enrichment analysis, and the results showed that CPLX1 was significantly and positively correlated with the “ERBB signaling pathway” (NES: 1.489, Nominal p-value: 0.021) and “Insulin signaling pathway (NES: 0.384, Nominal p-value: 0.014), which are able to promote the proliferation of cancer cells through the interaction of receptors (**Figures 7E, F**). In the GSE8671 dataset, CPLX1 was significantly positively correlated with the “Neurotrophin signaling pathway” (NES: -1.530, Nominal p-value: 0.021), “Oocyte meiosis” (NES: -1.512, Nominal p-value: 0.019) and “Progesterone mediated oocyte maturation” (NES: -1.669, Nominal p-value: 0.002) (**Figures 7G–I**). Interestingly, all three pathways are involved in the regulation of cell cycle, proliferation, survival and apoptosis in cancer, and their aberrant activation is closely related to tumor progression. However, the specific molecular mechanism by which CPLX1 promotes colorectal cancer development remains to be clarified by further experimental studies.

### 3.9 CPLX1 levels and immune cell infiltration

**Figure 8:**
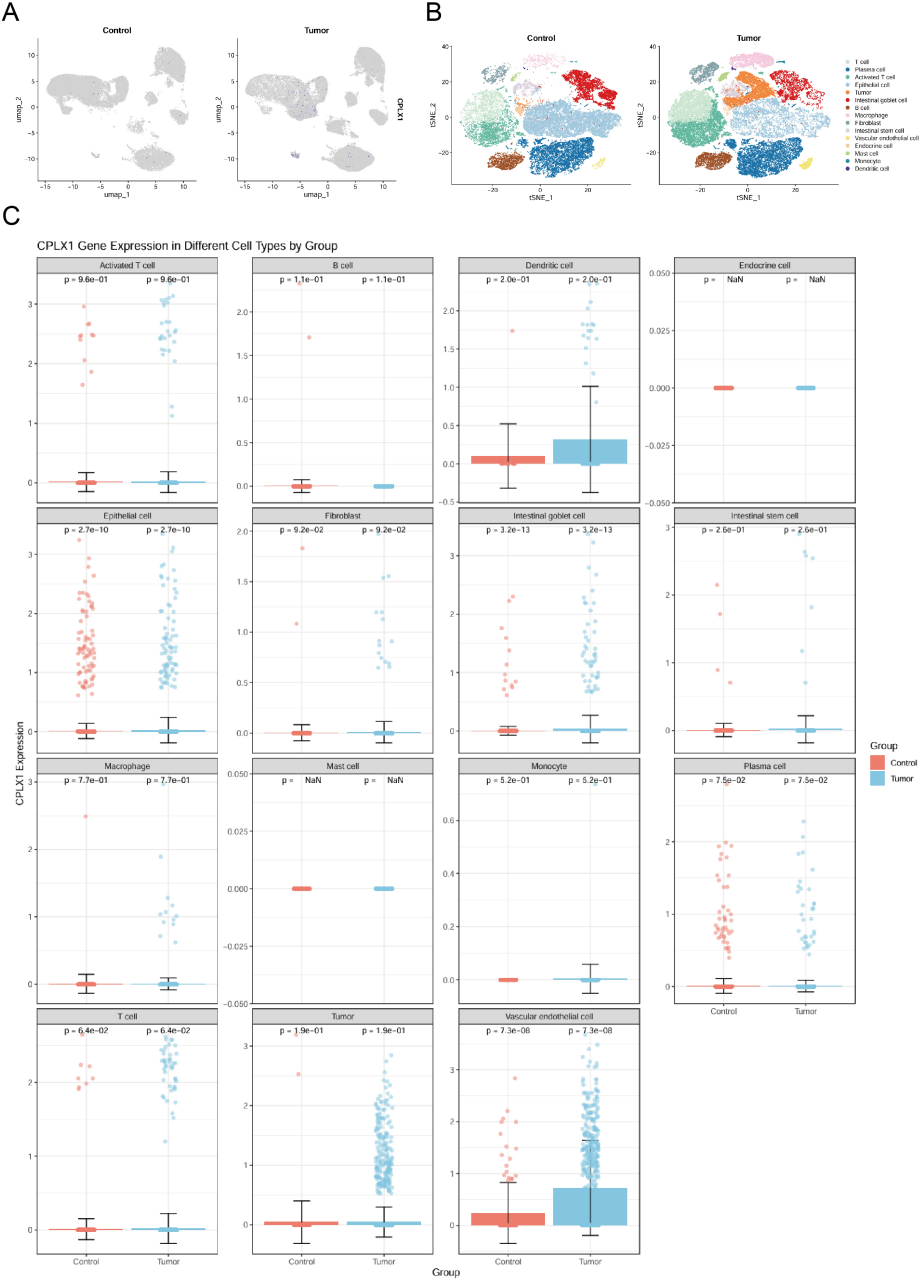
Analysis of CPLX1 changes and function at the single-cell level. **(A)** Single-cell level analysis of CPLX1 expression in control and tumor groups; **(B, C)** Single-cell level analysis of CPLX1 expression in different cellular fractions.

**Figure 9:**
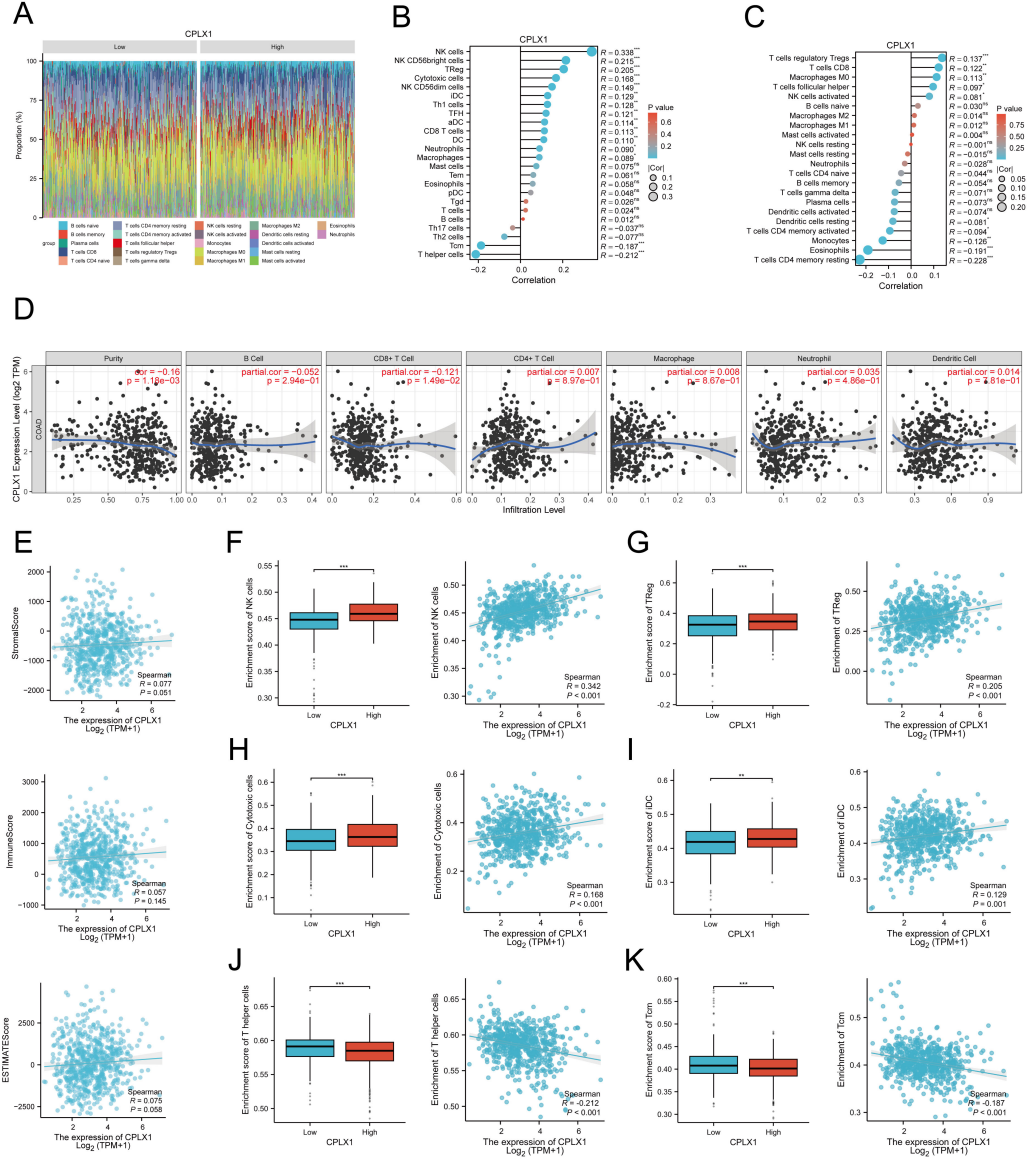
Associations between CPLX1 levels and immune cell infiltrative property within CRC. **(A)** Correlations across infiltration of 24 immune-cell-types and CPLX1 levels; **(B-D)** ssgsea **(B)**, CIBERSORT **(C)** and TIMER **(D)** to assess the correlation between infiltration of 24 immune cell types and CPLX1 levels; **(E)** StromalScore, ImmuneScore and ESTIMATEScore were also evaluated; **(F−K)** Degree of infiltration of NK cells **(B)**, TReg **(C)**, Cytotoxic cells **(D)**, iDC **(E)**, T helper cells **(F)** and Tcm **(G)** within high-/low-CPLX1 cohorts; CRC, colorectal cancer. *P<0.05; **P<0.01; ***P<0.001.

It has been shown that CPLX2 (which belongs to the same Complexin family as CPLX1) is expressed in B cells and has a regulatory role in immunoglobulin secretion (15). Although the role of CPLX1 in regulating the immune microenvironment has not been clearly demonstrated, considering the functional similarity between CPLX1 and CPLX2, we speculated that CPLX1 might play a role in regulating the immune microenvironment in colorectal cancer, and therefore we performed immune cell infiltration analysis for evaluation. We first downloaded GSE166555, a colorectal cancer single cell dataset, from the GEO database. Its processing revealed that CPLX1 was significantly higher expressed in tumor tissues than in the normal group (**Figure 8A**), and subsequent fractionation revealed its increased immune cell infiltration in tumor tissues (**Figure 8B**). The results of different cell subtypes were consistent with the previous ones (**Figure 8C**).We used CIBERSORT to detect the difference in immune cell infiltration in groups with different CPLX1 expression levels, and plotted an immune infiltration superimposed histogram (**Figure 9A**). CRC infiltration of 24 immune cell types was detected using ssGSEA (**Figure 9B**), CIBERSORT (**Figure 9C**) and TIMER (**Figure 9D**). StromalScore, ImmuneScore and ESTIMATEScore were also evaluated together (**Figure 9E**). The correlation between immune cells and CPLX1 levels was also assessed by Spearman’s correlation coefficient.CPLX1 was correlated with NK cells (R=0.342, *P*<0.001), TReg (R=0.205, *P*<0.001), Cytotoxic cells (R=0.168, *P*<0.001) and iDC (R=0.129, *P*<0.001) were positively correlated. In contrast, T helper cells (R=-0.212, *P*<0.001) and Tcm cells (R=-0.187, *P*<0.001) were negatively correlated with CPLX1 (**Figures 9F–K**).

### 3.10 Study of the correlation between CPLX1 and ferroptosis in CRC

**Figure 10:**
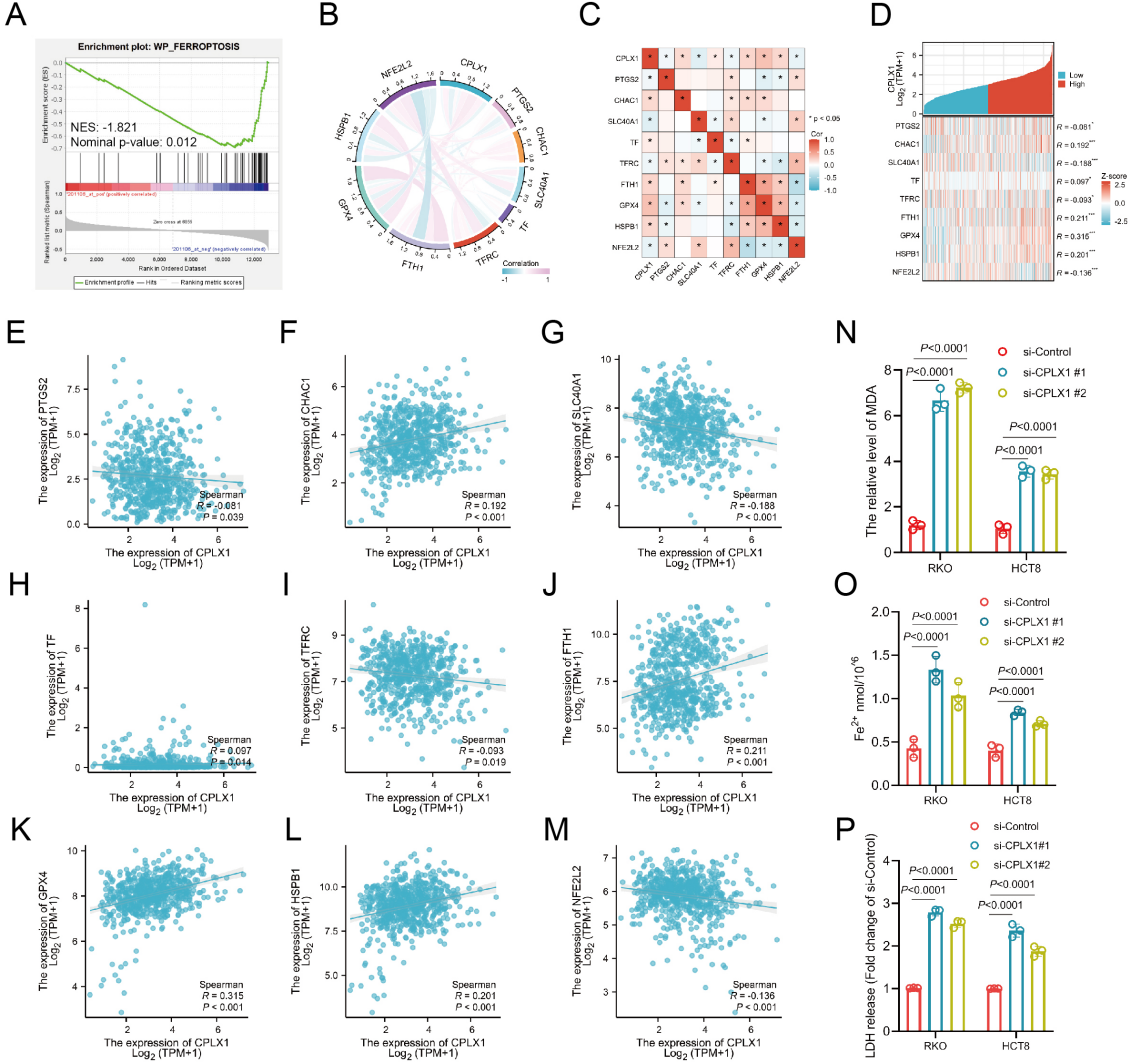
Relationships between expression of CPLX1 and that of ferroptosis-associated genes in CRC. **(A)** GSEA enrichment analysis of CPLX1 in relation to ferroptosis; **(B, C)** Correlations between CPLX1 and ferroptosis markers using TCGA data; **(D)** Correlations between CPLX1 and ferroptosis markers using GEPIA2 data; **(E-M)** Differentially expressed ferroptosis markers between high- and low-CPLX1 groups in CRC samples; **(N)** Detection of intracellular MDA content; **(O)** Detection of intracellular Fe^2+^ content CRC; **(P)** Detection of the release of LDH. colorectal cancer; TCGA, The Cancer Genome Atlas. *P<0.05; **P<0.01; ***P<0.001.

Ferroptosis was first described in 2012 (16), and unlike other death-related processes such as apoptosis and autophagy, it is iron-dependent and promoted by reactive oxygen species (ROS). It is associated with a variety of cellular changes, particularly in mitochondria, including cristae reduction, disruption of the outer mitochondrial membrane, and membrane compaction (17). Ferroptosis is regulated by multiple genes and pathways associated with cancer, suggesting that induction of ferroptosis may be a potential strategy to halt cancer progression. Importantly, ferroptosis is also involved in immunotherapy resistance in cancer (18). Possible mechanisms underlying the role of CPLX1 in colorectal cancer were analyzed by GSE41258, which revealed that the FERROPTOSIS pathway was significantly enriched and negatively correlated with the CPLX1 expression level (**Figure 10A**). Thus, we used the TCGA-COAD/READ dataset to assess the association between CPLX1 levels and ferroptosis, and showed significant associations between CPLX1 levels and ferroptosis markers including PTGS2, CHAC1, SLC40A1, TF, TFRC, GPX4, NFE2L2, HSPB1, and FTH1 (**Figures 10B, C**). TCGA-COAD/READ samples were categorized into low and high CPLX1 expression groups, and differentially expressed ferroptosis-related genes were identified (**Figure 10D**). The results revealed that the expression of CHAC1, TF, FTH1, GPX4, and HSPB1 was up-regulated in the high CPLX1 group (*P*<0.05), and the PTGS2, SLC40A1, TFRC and NFE2L2 expression level was reduced (*P*<0.05) (**Figures 10E–M**). Subsequently to validate the effect of CPLX1 on ferrroptosis in colorectal cancer cells at the cellular level, we examined the changes in MDA and Fe^2+^ content in two types of cells altered with CPLX1. It was found that knockdown of CPLX1 in both RKO and HCT8 cells resulted in upregulation of MDA, Fe^2+^ content and the release of LDH (**Figures 10N–P**).Thus, CPLX1 may influence CRC progression and prognosis through its ability to regulate ferroptosis.

### 3.11 CPLX1 regulates proliferation, migration and invasion of CRC cells

**Figure 11:**
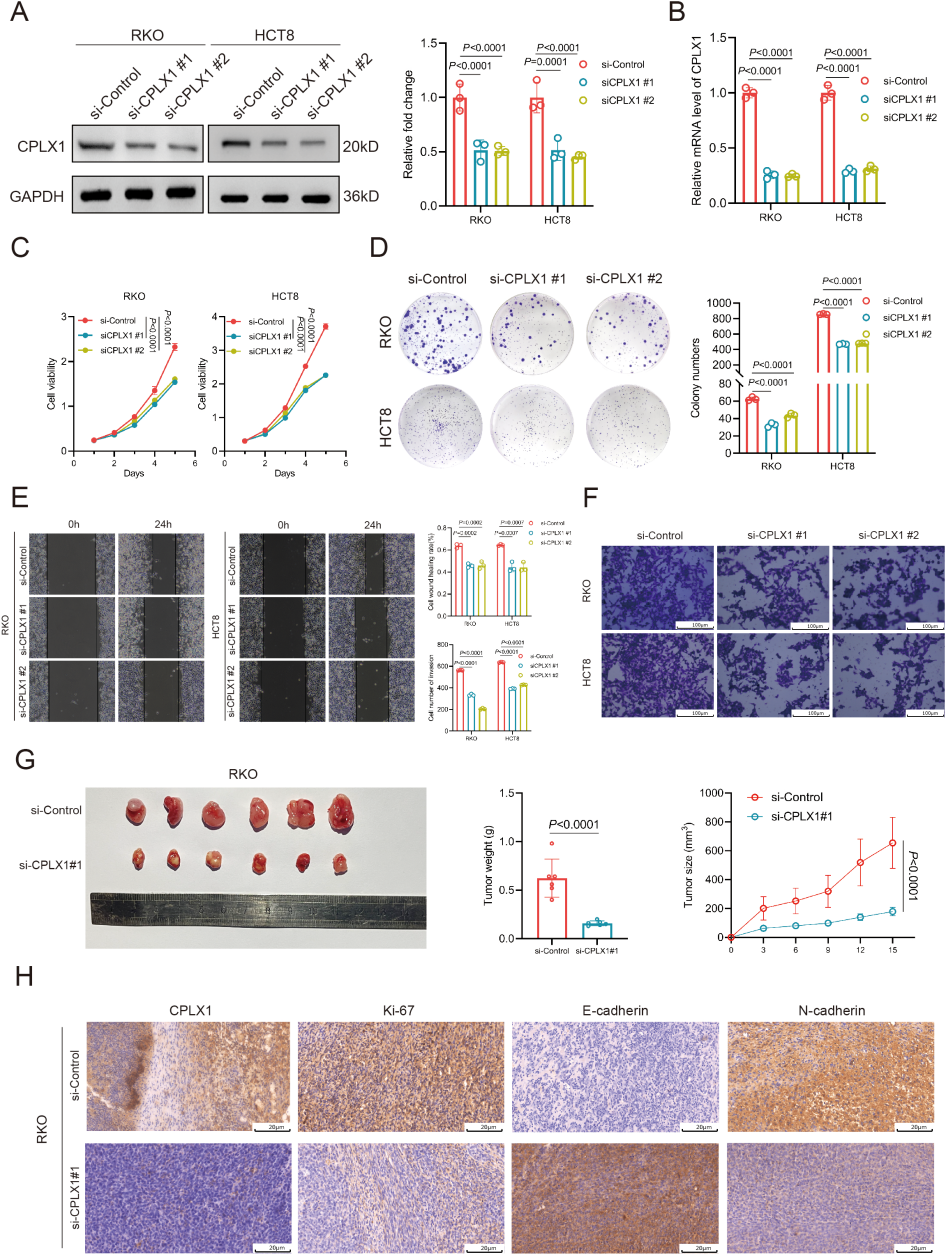
CPLX1 regulates proliferation, migration and invasion of CRC cells. **(A, B)** Down expressed CPLX1 in RKO and HCT8 cells was confirmed by Western blotting **(A)** and qRT-PCR **(B, C)** CCK-8 assay was used to detect the proliferation of RKO and HCT8 cells; **(D)** Colony formation assay was used to test the proliferative capacity of RKO and HCT8 cells; **(E)** Wound-healing assay was used to test the migratory capacity of RKO and HCT8 cells; **(F)** Transwell assay used to detect the invasive ability of RKO and HCT8 cells; **(G)** Effect of changes in CPLX1 on the tumorigenic capacity of RKO; **(H)** IHC detection of CPLX1, Ki-67, E-cadherin and N-cadherin expression.

To further evaluate the role of CPLX1, we knocked down CPLX1 in RKO and HCT8 cells and verified the knockdown efficiency by Western blotting and qRT-PCR (**Figures 11A, B**). CCK-8 assay showed that the cell viability was significantly reduced after CPLX1 knockdown (**Figure 11C**). The results of colony formation assay showed that the proliferation ability of cells was significantly reduced after knockdown of CPLX1(**Figure 11D**). Cell migration was detected by wound healing assay, and it was found that the migration ability of RKO and HCT8 cells was significantly reduced after knockdown of CPLX1 (**Figure 11E**). In addition, cell invasion was detected by Transwell assay, and it was found that the invasion ability of RKO and HCT8 cells was significantly reduced after knockdown of CPLX1 (**Figure 11F**). Then we injected two kinds of RKO cells subcutaneously in nude mice to observe the effect of CPLX1 alteration on the tumorigenic ability of RKO cells. The results showed that knockdown of CPLX1 could effectively reduce the tumorigenicity of RKO cells and inhibit the proliferation of tumor cells (**Figure 11G**). In addition, we examined the expression of CPLX1, the proliferation-related marker Ki-67 and the metastasis-related markers Eca and Nca in subcutaneous tumor tissues of both groups using immunohistochemical methods. The results showed that the expression of proliferation- and metastasis-related markers changed with the reduction of CPLX1 (**Figure 11H**). These results indicated that CPLX1 was involved in cell proliferation, migration and invasion of CRC cells.

## 4 Discussion

CPLX1 is gradually showing its importance in cancer research, and the multiple biological processes it participates in are closely related to tumorigenesis and development (3, 19, 20). Studies have shown that CPLX1 not only plays a key role in cell signaling, proliferation and apoptosis, but may also affect the formation of the tumor microenvironment (21–23). These findings provide new perspectives for our understanding of tumor biology and also open up new directions for early diagnosis and treatment of cancer. However, there is still some controversy about the role of CPLX1 in different types of cancer. On the one hand, some studies have pointed out that the high expression of CPLX1 is associated with the aggressiveness and poor prognosis of certain types of cancers (19); on the other hand, other studies have shown that it may have a tumor-suppressing effect under specific conditions (24). This diversity reflects the heterogeneity of tumors and the complexity of CPLX1 function. Therefore, future studies need to explore more systematically and in depth the specific mechanisms of CPLX1 in different tumor types and different microenvironments in order to better understand its dual roles in cancer. Looking forward, the potential of CPLX1 as a new target for cancer therapy is worth looking forward to (25). Through precise molecular targeting, interventions against CPLX1 may provide new therapeutic options for specific patients. In addition, combining CPLX1 with other therapeutic modalities is expected to enhance therapeutic efficacy.

Nevertheless, there is limited information regarding CPLX1 roles within CRC. Therefore, this investigation conducted a comprehensive bioinformatics-based analysis to explore its possible functional and diagnostic roles in this context. This investigation revealed that CPLX1 levels within tumor samples were markedly associated with tumor status and pathological phase, as well as survival outcomes. In addition, higher levels of CPLX1 expression were found to be related to worse patient prognosis and more advanced clinical stages of the tumor. Logistic regression analysis showed that CPLX1 levels were markedly associated with T pathologic stage, lymphatic invasion, as well as DSS and PFI event. Kaplan-Meier curves indicated that patient OS, DSS, and PFI were significantly reduced in patients with higher CPLX1 levels. Associations between CPLX1 and poor prognosis have been observed in various cancer types (19, 20, 26, 27). Such dataset outcomes imply that CPLX1 could be a prognostic biomarker within numerous cancers, including CRC. Further investigations into possible CPLX1 roles within CRC using annotation/pathway analyses indicated its involvement in various pathways, including pattern specification process and membrane microdomain, suggesting that CPLX1 may modulate the organization of the extracellular structures of tumor cells. The importance of genetic and epigenetic contributions to cancer development is well-known. For instance, mutations in the immune checkpoint gene PD-L1 influence its structure, cellular expression, and overall functioning (28). Overexpression of the JAK2/PDL1/PD-L2 pathway leads to activation and altered functioning of other immune checkpoint molecules (29). However, it was found that CPLX1 was mutated in only 0.03% of CRC tissue samples, nor were mutations linked with OS and DSS in CRC. The tumor microenvironment consists not only of tumor cells but also includes a variety of immune and stromal cells, including fibroblasts, endothelial cells, and neurons (30). Immune cellular infiltration types/degrees can assist in predicting the patient’s response to immunotherapy. Here, we identified an association between infiltration and CPLX1 levels, observing that CPLX1 was negatively associated with the numbers of both Tcm and T helper cells in CRC tumor samples. CPLX1 levels were also identified as positively linked to the levels of NK cells and TReg, suggesting that it may affect the composition of the tumor microenvironment through various mechanisms. Our findings thus suggest that elevated levels of CPLX1 are closely associated with mechanisms promoting immune escape in CRC tumor cells, thus contributing to both tumor growth and progression. Targeting of ferroptosis has been suggested for treating cancer, especially for the treatment of refractory tumors (16). A number of tumor suppressor proteins, such as P53, fumarase, and BAP1, can sensitize tumor cells to ferroptosis (31). Here, the relationships between CPLX1 levels and ferroptosis-associated genes were investigated, and positive correlations were observed between them. These results indicate that the promotion of tumorigenesis by CPLX1 is associated with the ability of CPLX1 to regulate ferroptosis and may provide a new direction in targeting ferroptosis for treating CRC.

To our knowledge, this study probes links across CPLX1 and outcomes in patients with CRC. Nevertheless, there are certain limitations. First, the analyses were bioinformatics-based, and experimental verification is necessary. Furthermore, the numbers of CRC patients and controls differed, as did the type of interventions used. Further verification and investigation are thus required.

Although we have made some preliminary findings, more in-depth and systematic research work is still needed to more comprehensively understand the specific application value of CPLX1 in colorectal cancer treatment. Future research directions should be more focused on exploring how to effectively integrate the assessment results of CPLX1 into clinical practice, in the expectation of significantly improving the prognosis of colorectal cancer patients and increasing their survival rates, thus bringing better quality of life and hope to patients. Firstly, conduct *in vitro* and *in vivo* experiments to elucidate the specific mechanism of CPLX1 in CRC cell proliferation, migration, and invasion. This will provide a deeper understanding of its biological significance in CRC progression. Secondly, investigate the mechanisms by which CPLX1 regulates ferroptosis. Our study found a positive correlation between CPLX1 levels and ferroptosis-related genes, but the underlying pathways remain unclear. Future research should explore how CPLX1 influences iron metabolism and lipid peroxidation to modulate ferroptosis. Lastly, assess the potential of CPLX1 as a therapeutic target. Although CPLX1 has been implicated in the prognosis of various cancers, its therapeutic potential remains underexplored. Evaluating the efficacy of antibodies or small-molecule inhibitors targeting CPLX1 in CRC treatment could provide new therapeutic strategies.

In conclusion, CPLX1 holds promise as a biomarker and potential therapeutic target in CRC. Future studies should focus on elucidating the biofunctional mechanism of action of CPLX1, its role in ferroptosis, and its therapeutic potential. Addressing these questions will provide valuable insights and may lead to new diagnostic and therapeutic strategies for CRC patients.

## 5 Conclusions

It was found that CPLX1 is upregulated in CRC tissue samples and that this was associated with reduced patient survival. Functional analysis suggested that CPLX1 may mediate CRC progression by modulating the function of extracellular membranes. Such revelations imply that CPLX1 could be a possible biomarker for CRC diagnoses and prognoses.

## Data Availability

The original contributions presented in the study are included in the article/supplementary material. Further inquiries can be directed to the corresponding authors.
